# An intervention study on the secondary prevention medication adherence of ischemic stroke patients based on the protection motivation theory combined with medication literacy education in the AI-HEALS: a randomized controlled trial protocol

**DOI:** 10.3389/fpubh.2025.1677253

**Published:** 2026-01-29

**Authors:** MaoDa Teng, JieMei Wei, Yang Jiang, XiaoXiao Guo, Mei Zhao, Lei Shi, XiaoMing Zhou, Niuniu Sun

**Affiliations:** 1 School of Nursing, Henan University of Science and Technology, Luoyang, Henan, China; 2 Linyi Central Hospital, Linyi, Shandong, China; 3 Jitang College, North China University of Science and Technology, Tangshan, Hebei, China; 4 Shandong Provincial Hospital Affiliated to Shandong First Medical University, Jinan, China; 5 Department of Pharmacy, Dongying People’s Hospital, Dongying, Shandong, China

**Keywords:** AI-HEALS, drug literacy, ischaemic stroke, medication adherence, PMT, protective motivation theory, secondary prevention

## Abstract

**Background:**

This study aims to improve medication adherence in ischemic stroke (IS) patients using a dual-driven motivation-competence intervention framework based on the theory of protective motivation (PMT) and the medication literacy model. Therefore, this study used the WeChat platform as the intervention vehicle and innovatively introduced the AI-HEALS, including the AI intelligent question and answer system and the HEALS (Health Education Accurately Linking System), aiming to achieve personalised intervention through the intelligent terminal.

**Methods:**

The study will conduct a single-blind, single-centre randomised controlled trial in Linyi City Central Hospital, Shandong Province, with IS patients over 18 years old. The intervention group will receive routine care combined with the AI-HEALS, while the control group will receive routine care alone. Primary outcomes include changes in medication adherence levels at baseline and 1, 3, and 6 months of follow-up. Secondary outcomes encompass medication behavior management, medication literacy, self-efficacy, social cognition, psychological levels, and clinical outcome indicators.

**Discussion:**

The aim of this study was to develop an intervention programme for secondary prevention medication adherence in patients with IS by combining PMT and medication literacy models and using the AI-HEALS. Although PMT has shown effectiveness in improving health behaviors in patients with chronic diseases, systematic studies of its application to stroke patients are lacking. Our intervention framework aims to stimulate motivation, develop competence, and solidify behaviors to overcome the limitations of a single theory in complex medication scenarios. The AI-HEALS addresses the challenges of traditional mHealth interventions and is expected to improve patients’ medication literacy and adherence through precise knowledge push, dynamic interaction support, and full-cycle management. The development of the AI-HEALS helps to reduce the burden on healthcare professionals, improve the distribution of healthcare resources, and have potential health economics benefits.

**Clinical trial registration:**

https://www.chictr.org.cn, ChiCTR2500101457.

## 1 Introduction

Stroke is an acute cerebrovascular disease that manifests itself as a sudden neurological impairment and can be fatal in severe cases (1). It is characterised by high morbidity, high mortality, high disability, high recurrence and high economic burden. In China, the morbidity, mortality and disability rates of stroke are higher than those in many developed countries, and the mortality rate ranks first among all diseases (2). Strokes are classified into ischemic stroke (IS), haemorrhagic stroke and subarachnoid haemorrhage, of which IS is the most common, accounting for about 73.33% of all strokes (3). During an acute attack of IS, the patient often suffers from hemiparesis, hemiplegia, hemianopsia, hemianopic blindness and aphasia, and some of the patients may also suffer from headache, vomiting and impaired consciousness. The risk of IS increases significantly with age, and doubles with every 10-year increase in the age of 55 years (4). The risk of stroke increases with age, and doubles with every 10-year increase in the age of 55 years (5). The risk of IS increases significantly with age, doubling with every 10-year increase after 55 years of age (4). The prevalence of IS is expected to continue to rise over the next 20 years as the population ages (6). Studies have predicted that the global prevalence of IS may reach 89.32 per 100,000 person-years by 2030 (6), which has become a major health challenge in China and globally.

In the early stage of IS, intravenous thrombolysis (IVT) is an effective treatment to restore cerebral blood flow and improve neurological function, but it cannot prevent cerebral atherosclerosis or eliminate the risk of recurrence. Some patients still have high rates of disability and recurrence after thrombolysis due to insufficient self-management and treatment compliance (7). Therefore, the implementation of secondary prevention measures is essential to reduce the recurrence rate and improve the prognosis. Secondary prevention targets patients who have had a stroke and aims to reduce the risk of recurrence and improve prognosis (8, 9). In the United States, approximately 700,000 ischemic strokes occur each year, a quarter of which are recurrent strokes, and the risk of death in patients with recurrent strokes is twice as high as that in patients with first-time strokes (10–12). Patients who have had a stroke and TIA have a 40% probability of having another stroke within 5 years and are at high risk for recurrent stroke (13). Most risk factors (e.g., hypertension, diabetes mellitus, hyperlipidemia, etc.) are preventable and controllable, so early secondary prevention is of great significance in improving prognosis (14). Countries have adopted a series of secondary prevention measures, including vascular risk factor management (e.g., antihypertensive, hypoglycemic, and lipid-lowering treatments) (15–19), and lifestyle interventions (e.g., Mediterranean diet, regular exercise, and smoking cessation) (20–22), which are effective in reducing recurrence rates and improving the quality of life of patients.

In this study, medication adherence is defined as the patient’s active choice and commitment to consistently follow their prescribed treatment regimen, including the correct frequency, dose, and route of administration. This concept emphasizes the patient’s voluntary role and responsibility for their own well-being, distinguishing it from the more passive notion of compliance. In secondary stroke prevention, medication adherence is key to preventing recurrence, but patient adherence is generally poor and declines over time. Data from a Swedish study showed that the proportion of patients adhering to antithrombotic medication was 86.3% 1 year after discharge, decreasing to 74.2% after 2 years (23); in China, more than one-third of patients did not use secondary prevention medication for a long period of time, and 30 to 60% of patients had low rates of knowledge, treatment, and control of stroke risk factors (24). Several studies have shown that patients’ medication adherence is poor after discharge from the hospital and that poor adherence is an independent risk factor for poor prognosis (25–28). Several intervention studies have been conducted at home and abroad to improve medication adherence, such as motivational interviewing, community nursing interventions, and smartphone apps (29–31), all of which have achieved certain results. In China, cognitive education interventions, tri-partite medication reorganisation programmes, and the ‘Seven Ones’ interventions by brain-centre health managers have also been effective in improving medication adherence (32, 33). IS patients often have a combination of chronic diseases and require multiple medications (e.g., antiplatelet agents, anticoagulants, antihypertensives, etc.), which increases the difficulty of managing medication behaviors and the need for medication management. Difficulty in managing medication-taking behaviors and the risk of medication errors, leading to a further decline in adherence (34–36). Therefore, it is particularly important to increase the level of patients’ medication literacy to improve medication adherence and overall outcomes.

Protection Motivation Theory (PMT) is a psychological theory used to explain the adoption of protective behaviors by individuals (37), proposed by Rogers in 1975 (38) and refined in 1983 (39). The theory suggests that protective behaviors are determined by a combined assessment of threat and coping, comprising three components: information sources, cognitive mediators, and coping modes. In particular, threat assessment includes perceived susceptibility, perceived severity, external rewards, and internal rewards; and coping assessment includes self-efficacy, response efficacy, and response cost. Perceived susceptibility, severity, response efficacy, and self-efficacy promote health behaviors, while external rewards, internal rewards, and response costs impede health behaviors. PMT has been widely used for disease management and health behavior change, such as prevention of skin cancer, reduction of e-cigarette smoking behaviors, and improvement of oral health knowledge (40–44). In China, PMT has also been applied in the areas of AIDS prevention and treatment, sedentary behavioral interventions for patients with coronary heart disease, and has effectively improved patients’ health behaviors and self-efficacy (45, 46).

Drug literacy refers to the ability of patients to acquire, understand, communicate, calculate and process drug information, and make correct medication decisions accordingly. The concept was proposed by Raynor in 2008 and later refined by Pouliot et al. (47, 48). Drug literacy consists of four dimensions of functional, communicative, critical and computational literacy, and five sub-dimensions of accessing, understanding, evaluating, computing and communicating drug information. Drug literacy is influenced by a combination of social, personal and health system factors (49). The conceptual model of drug literacy can significantly improve patients’ medication adherence and quality of life and reduce medication errors (50, 51). Overseas studies have improved patients’ medication literacy and adherence through hieroglyphic instructions and mobile health services (52–56). In China, drug literacy theory has been applied to interventions for patients with hypertension and myocardial infarction, effectively improving patients’ drug literacy and medication adherence (57, 58). However, there are still fewer studies on drug literacy for IS patients.

Previous studies have found that interventions to enhance patient medication adherence include patient education and health literacy promotion, pharmacist-led interventions, digital health technologies, multidisciplinary teamwork and behavioral theory-driven interventions, but these measures have limitations such as short follow-up, insufficient effect on patients with low education levels and multiple medication use, potential to exacerbate health inequalities, and insufficient economic evaluation (59, 60). Therefore, there is a need to develop more optimal strategies that incorporate patient characteristics, and multimodal interventions (PMT theory + Digital technology + Multidisciplinary collaboration) deserve further exploration.

**Figure 1:**
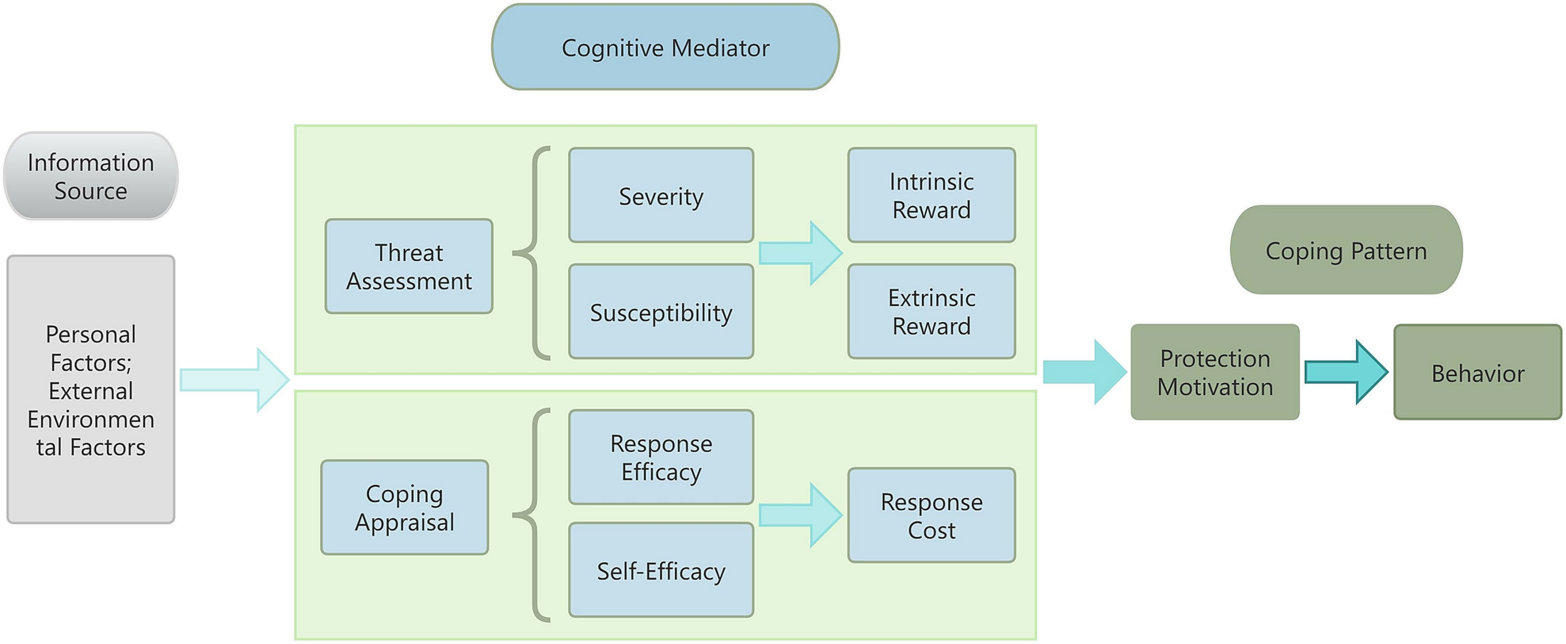
PMT integration theory diagram.

In this study, a theoretical framework for secondary prevention medication adherence in patients with IS has been constructed and practically tested by combining Protective Motivation Theory (PMT) and a conceptual model of drug literacy (Figure 1). In this context, this study will combine the Artificial Intelligence-Health Education Accurately Linking System (AI-HEALS) intervention system (61), i.e., through the WeChat platform, to construct the “Internet + Healthcare Education “model through the WeChat platform to provide efficient solutions for IS patients. The system integrates the functions of medication consultation, health knowledge education, behavioral detection, psychological support and health reminder, and is divided into the AI intelligent Q&A system and the HEALS. The AI intelligent Q&A system will be based on the information finely scrutinised by the clinicians and pharmacists, constructed with the medical professional AI intelligent body (AI Agent), and use the large language model (deep seek) to answer the patient’s questions accurately. Provide accurate and professional medication counselling; the HEALS will make use of the convenience of the Internet to make up for the lack of traditional nursing interventions, to meet patients’ needs and to improve health outcomes. The platform constructed in this study combines the capabilities of the big language model and the HEALS, as well as the professional medication knowledge base and stroke health knowledge, to provide patients with scientific and intelligent health education services, which is expected to improve patients’ medication adherence and health management ability.

This study aims to improve the secondary prevention medication adherence of IS patients through the intervention model of PMT combined with drug literacy education. The specific objectives are as follows:

1. To construct a secondary prevention medication adherence intervention programme for IS patients based on the PMT and drug literacy model, and to validate its effectiveness in improving medication adherence, self-efficacy for medication administration, drug literacy and clinical indicators in IS patients.
2. Evaluate the effectiveness of the AI-HEALS in improving the self-efficacy of health behaviors, quality of life and psychological status of IS patients, and track the reduction in the risk of relapse and readmission.

## 2 Methods

### 2.1 Study design

**Figure 2:**
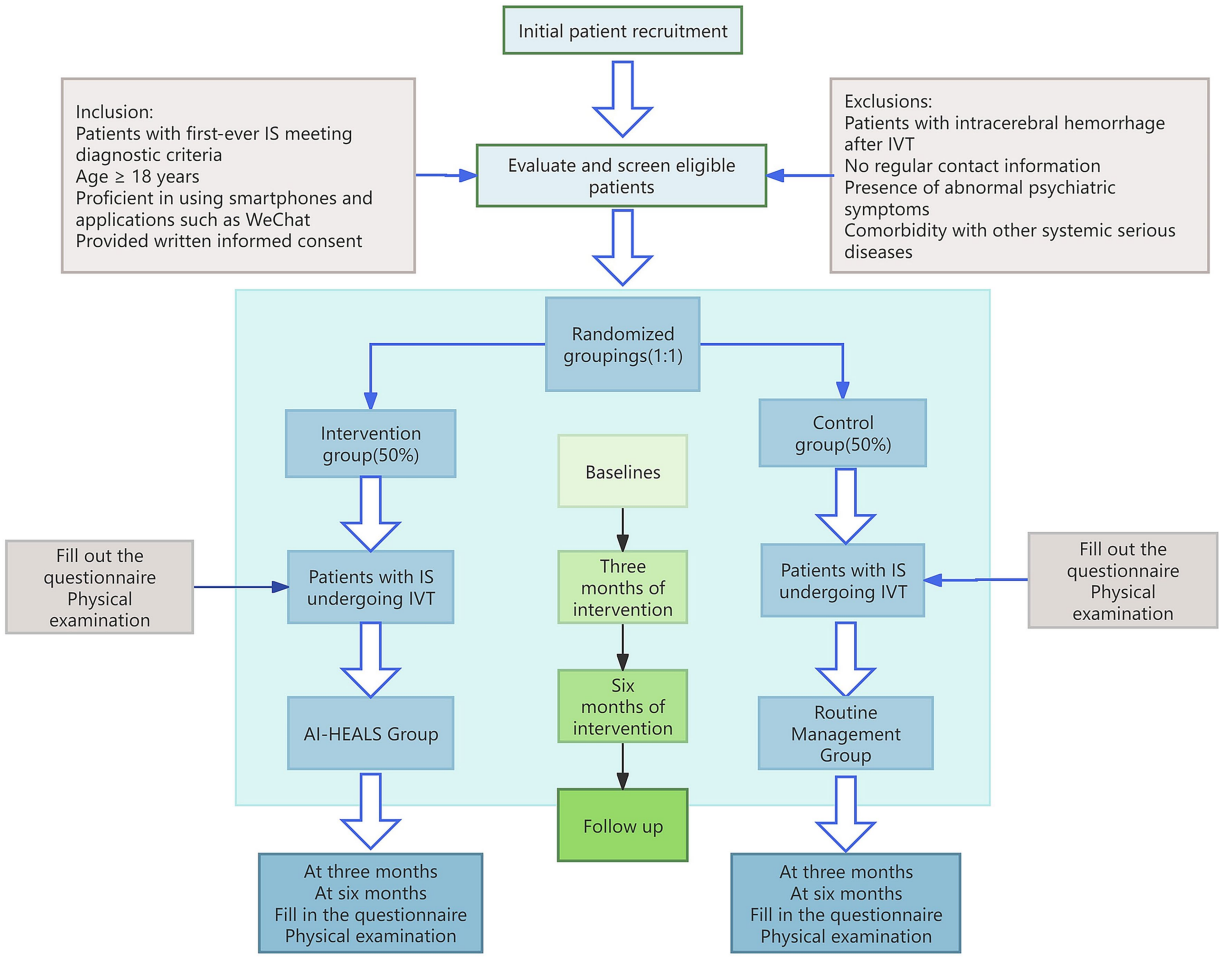
Flowchart of patient recruitment and study implementation.

This study is a hospital-based randomised controlled trial designed to compare the effectiveness of an intervention based on the AI-HEALS’s theory of protective motivation combined with drug literacy education with a usual care control group. The study will use quantitative methods, measured by clinical reagent tests and internationally used scales to ensure the objectivity of the trial. The study period is scheduled from June to December 2025. The control group will receive standard care, including admission education, dietary and psychological care, medication and rehabilitation care, life care, health counselling, and prognostic guidance. The intervention group will receive the AI-HEALS intervention for 3 months on the same basis. Patients will undergo questionnaires and physical examination at baseline, 3 months and 6 months post-intervention to assess changes in self medication behavior management, social cognitive and psychological indices as well as clinical health indice. The flow of the study is shown in Figure 2 and Table 1.

**Table 1:** Timetable for study enrolment, intervention and evaluation.

Study period		Recruitment	Allocation	Intervention		Follow-up
Time point		0 M	0 M	0 M	3 M	6 M
Time point	Eligbility screen	√				
	Informed consent	√				
	Allocation		√			
Intervention	PMT Management			√	√	
	Control					
Assessments	Sociodemographic variables			√		
	Disease-related materials			√		
	Clinical Health Indicators			√	√	√
	Medication Self-Management			√	√	√
	Social Cognition and Psychology			√	√	√

### 2.2 Study setting and randomization

The study remained single-blind, with participants unaware of their group allocation. All outcome assessors, including the triage nurses and laboratory staff, were blinded to the group assignments to minimise measurement bias. Data were collected and analysed by an independent third-party researcher and anonymised and coded to reduce measurement bias. Physiological outcome indicators were assessed at outpatient review by blinded triage nurses and laboratory staff who were unaware of subgroup information. The study population was screened from patients admitted to the inpatient Neurology Department between June and December 2025, following the inclusion and exclusion criteria, and the study will be conducted in the Neurology Department of Linyi Central Hospital, Shandong Province, China, in the first and second wards.

Eligible participants were randomly allocated to either the intervention group or the control group in a 1:1 ratio. The randomization sequence was generated by an independent biostatistician using a computer-based random number generator in SPSS (version 27.0, IBM Corp.). To ensure balance in group sizes over time, block randomization with a fixed block size of 4 was utilized. To control for potential baseline imbalances in key prognostic factors, the randomization was stratified by baseline NIHSS score (≤5 vs. > 5).

Allocation concealment was guaranteed to prevent selection bias. The randomization assignments were stored in sequentially numbered, opaque, sealed envelopes (SNOSE). These envelopes were prepared and held by an independent research coordinator who was not involved in the recruitment or assessment of participants. After a participant’s eligibility was confirmed and baseline data were collected, the recruiter would open the next envelope in sequence to determine the participant’s group assignment.

### 2.3 Study participants

IS patients admitted to Linyi Central Hospital in Shandong Province between June and December 2025 were selected as participants for this study. Eligibility was verified through a two-step process. First, trained staff screened electronic medical records of consecutive ischemic stroke patients. Second, a board-certified neurologist confirmed eligibility through a clinical assessment. Informed consent was obtained only after this verification was complete. Upon admission, patients will complete trial enrolment and sign a study informed consent form via face-to-face, followed by initiation of the 3-month intervention. Although participants are not recruited or initiating the intervention simultaneously, they will all follow the same intervention cycle.

To minimise the risk of cross-group contamination, we implemented stringent control measures across three dimensions: physical, personnel, and procedural. Physically, we allocated distinct follow-up times and consultation rooms for each group, with intervention materials kept under lock and key. Personnel-wise, all staff underwent standardised training, were explicitly prohibited from mentioning intervention details to the control group, and were provided with standardised response scripts. Procedurally, we implemented clear role-based segregation, advised patients at enrolment to avoid cross-group communication, and ensured effective execution of all isolation protocols through regular monitoring.

The study has been approved by the Ethics Committee of Linyi Central Hospital in Shandong Province (LCH-LW-2025031) and registration of the study protocol has been completed with the China Clinical Trial Registry (registration number: ChiCTR2500101457).

Inclusion criteria:

1. Meet the diagnostic criteria for first-episode ischaemic stroke (62);
2. Age 18 years and above;
3. Be able to operate smartphones and social media applications such as WeChat;
4. Patients or family members signing an informed consent form.

Exclusion criteria:

1. Patients with cerebral haemorrhage after intravenous thrombolytic therapy;
2. Patients with indications for mechanical thrombolysis by cerebral angiography (CTA) or other imaging tests (63, 64);
3. Patients with severe mental illness, impaired consciousness, and inability to communicate effectively;
4. Patients with severe gastrointestinal bleeding or serious cardiac, hepatic, or renal insufficiency;
5. Patients with cancer or haemorrhagic disease.

### 2.4 Sample size

The sample size was calculated *a priori* using G*Power software (version 3.1.9.7) for a two-tailed independent-samples t-test. The primary outcome was the difference in the Adherence to Refills and Medications Scale (ARMS-7) score between the intervention and control groups at the end of the study period. The following parameters were used for the calculation: Significance Level (*α*): A two-tailed α of 0.05 was considered statistically significant. Statistical Power (1-*β*): The desired power (1-β) was set at 0.80. Effect Size (Cohen’s d): Based on a review of similar literature (65, 66), we hypothesised a mean ARMS-7 score of 10.0 (SD = 2.0) in the control group and 9.0 (SD = 2.0) in the intervention group. This corresponds to a mean difference of 1.0 and a pooled standard deviation of 2.0, yielding a medium effect size (Cohen’s d) of 0.5. The sample size was calculated using the following formula:


$$n={\left(\frac{Z_{\alpha /2}+Z_{\beta }}{\Delta /\sigma }\right)}^{2}$$

Using these parameters (*α* = 0.05, power = 0.80, effect size d = 0.5), the required sample size was determined to be 32 participants per group.

To compensate for potential participant dropouts and ensure adequate power for the final analysis, we inflated the sample size by an estimated attrition rate of 20%. This resulted in a final target recruitment of 40 participants per group, for a total sample size of 80 stroke patients.

### 2.5 Interventions

**Figure 3:**
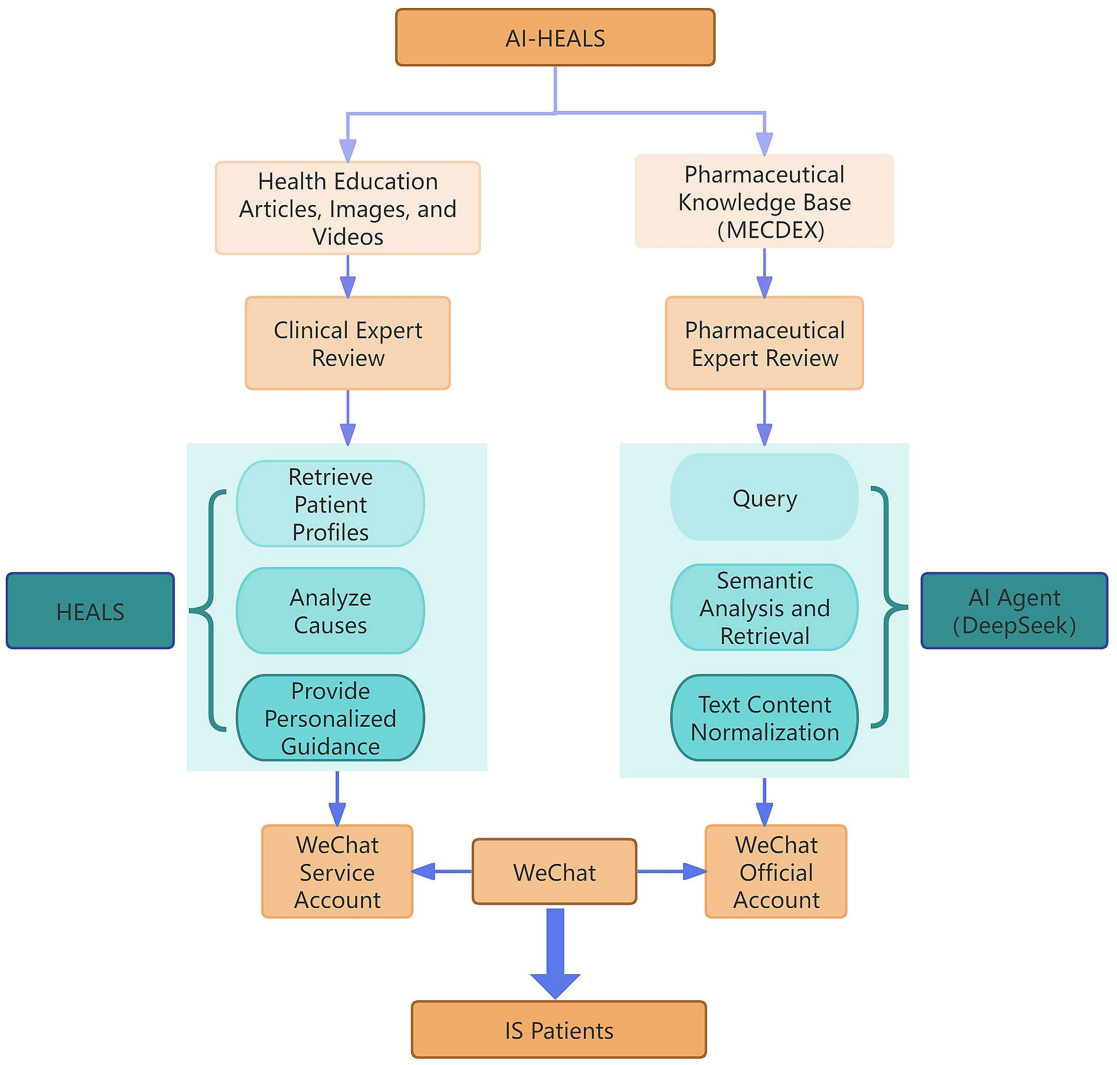
Schematic diagram of the operation of the intervention system.

The study was divided into a control group and an intervention group, the control group received routine medical care, and the intervention group received an educational intervention based on the AI-HEALS of protective motivation theory combined with drug literacy, and the content of the intervention was designed based on the conceptual framework of the protective motivation theory combined with drug literacy, and the content of the intervention can be found in Supplementary material, and the operation diagram of the intervention system can be found in Figure 3.

The intervention comprises a three-stage core educational programme. The first session is delivered within 48 h of admission (approximately 30 min), the second is scheduled 24–48 h prior to discharge (approximately 30 min), and the third involves remote intervention via the AI-HEALS system following discharge (continuing for approximately 3 months). The intervention is delivered using a hybrid model. The initial two in-hospital sessions comprise face-to-face, one-to-one sessions between nurses and patients, utilising mobile educational services provided by the AI-HEALS system. The post-discharge session is delivered remotely via the AI-HEALS mobile application. All neurological nurses responsible for delivering the intervention underwent mandatory standardised training led by the Head Nurse and neurological physicians. This four-hour training covered the research protocol, theoretical foundations of the intervention, and operational protocols for the AI-HEALS system. To ensure intervention fidelity, the Head Nurse conducted weekly randomised spot checks of 10% of sessions via session recordings and direct observation to verify protocol adherence. To consolidate learning outcomes, the AI-HEALS system automatically delivered daily push notifications containing key medication reminders and health alerts to the intervention group for 3 months post-discharge (see Supplementary material).

## 3 Outcomes

Primary Outcome: Change in medication adherence from baseline and at 3 and 6 months of follow-up.

Secondary outcomes:

1. Changes in medication self-behavior: medication literacy levels, correct medication taking, self-efficacy for rational medication use.
2. Social cognitive and psychological: quality of life, depression, anxiety.
3. Clinical health indicators: blood pressure (BP), low-density lipoprotein (LDL-C) level, glycated Hemoglobin (HbA1c), fasting plasma glucose (FPG), readmission rate.

The corresponding study questionnaires completed by all patients at baseline and follow-up were hosted on a web-based platform (Questionnaire Star). The questionnaires were distributed by the researchers through Questionnaire Star, and patients were instructed to complete them by the researchers either face-to-face (during hospitalisation) or by telephone (after discharge). Patient demographic information was collected through the case system and self-designed questionnaires.

## 4 Variables measurement

Socio-demographic variables will be collected from patients at baseline. Clinical reagent testing and questionnaires will be completed at baseline as well as at 3 and 6 months of follow-up, with data collection times shown in Table 2.

**Table 2:** Data collection components and collection timeline.

Data collection component		Timepoint		
		0 M	3 M	6 M
Sociodemographic variables	Age, gender, educational level, economic status, living conditions ; Duration of illness, number of medications taken, history of adverse drug reactions, source of drug information	√		
Clinical Health Indicators	BP, LDL-C, HbA1c, FPG, NIHSS	√	√	√
Medication adherence	Questionnaire: ARMS-7	√	√	√
Quality of life	Questionnaire: EuroQol-5 Dimensions 5 Levels(EQ-5D-5L)	√	√	√
Medication Literacy	ML-scale	√	√	√
Medication administration accuracy rate	Questionnaire: Medication Error Survey Form	√	√	√
Self-efficacy for Appropriate Medication	Questionnaire: Self-efficacy for Appropriate Medication Use Scale, SEAMS	√	√	√
Depression and anxiety	Questionnaire: Simplified version of the Anxiety Depression Scale (PHQ-4)	√	√	√
Readmission rate	Readmission rate			√

## 5 Questionnaires

### 5.1 Refill or medication adherence scale (ARMS-7)

ARMS-7was used to assess the level of medication adherence in patients. The scale was developed by Kripalani et al. (67) and consists of seven entries divided into two dimensions: medication adherence (entries 1 to 4) and refill behavior (entries 5 to 7). Each entry is scored on a 4-point Likert scale, where 1 represents ‘not at all’ and 4 represents ‘always’, with a total score ranging from 7 to 28. It is important to note that the 7th entry is reverse scored, i.e., 1 for ‘always’ and 4 for ‘not at all’. Higher total scores indicate poorer medication adherence, and the ARMS-7 scale has high reliability and validity, with item correlation coefficients ranging from 0.35 to 0.58 and Cronbach’s alpha coefficients of 0.75, which suggests that the scale has good internal consistency (64).

### 5.2 Simplified version of the medication literacy scale (ML-scale)

A simplified version of the Medication Literacy Scale (ML-Scale) previously validated by our group was used to assess the level of drug literacy in patients (68). The scale was previously validated by our group and was highly compatible with the present study. The reliability of the scale was confirmed by Cronbach’s alpha coefficient (0.799) and Omega coefficient (0.785), indicating good internal consistency of the assessment tool. The results of the validation factor analysis showed that the average variance extracted (AVE) ranged from 0.718 to 0.830 and the composite reliability (CR) ranged from 0.835 to 0.830, which are data that indicate that the convergent validity of the scale is more satisfactory. Structural validity was supported by the following metrics: chi-square to degrees of freedom ratio (/df) of 53.877, goodness-of-fit index (GFI) of 0.996, canonical fit index (NFI) of 0.997, relative fit index (RFI) of 0.992, and root-mean-square error approximation (RMSEA) of 0.042. The scales were scored on a five-point Likert scale, with a total score range from 0 to 30, with higher scores indicating a higher level of drug literacy in the patient.。.

### 5.3 Medication error questionnaire

In this study, the Medication Error Questionnaire was used to assess the errors that patients may have made during the actual administration of medication. The questionnaire was designed by Huiling (69) and contained 10 entries covering various aspects of medication errors. Each entry was also scored on a 4-point scale with a total score ranging from 10 to 40, with higher scores indicating a lower incidence of medication errors. The Cronbach’s alpha coefficient was 0.852, indicating good internal consistency of the scale.

### 5.4 Self-efficacy for appropriate medication use scale (SEAMS)

This scale was developed by Risser et al. (70) in 2007 based on the self-efficacy theory to assess patients’ self-efficacy in appropriate medication use. The scale consists of two dimensions, confidence in adhering to medication in uncertain situations and difficult situations, and consists of a total of 13 entries. A Likert 3-point scale was used, with scores ranging from 1 (no confidence) to 3 (very confident) and a total score ranging from 13 to 39, with higher scores indicating higher levels of self-efficacy in rational medication use among the subjects. The scale was rigorously translated by Dong (71) based on Brislin’s translation principles and was investigated in 480 stroke patients to test its reliability and validity. The retest reliability of the Chinese version of the SEAMS was 0.932, and the Cronbach’s alpha coefficient for the whole scale was 0.934. Two cofactors were extracted by exploratory factor analysis, with a cumulative variance contribution of 55.821%, and the factor loadings of all the entries on their communal factors exceeded 0.4. The content validity index at the scale level (CVI) was 0.913, indicating that the scale is applicable and has good applicability for the evaluation of medication taking in stroke patients.

### 5.5 Quality of life EQ-5D-5L (EuroQol-5 dimensions 5 levels)

The quality of life of patients was assessed using the EQ-5D-5L, an improved version of the EQ-5D scale, which adds five levels to the original five dimensions (72, 73), namely, ‘no difficulty’, ‘minor difficulty’, ‘moderate difficulty’, ‘major difficulty’ and ‘complete difficulty’. ‘minor difficulty’, ‘moderate difficulty’, ‘major difficulty’ and ‘total difficulty’. This enables a more detailed assessment of health status. Compared to the original EQ-5D (i.e., EQ-5D-3L), the EQ-5D-5L maintains consistency in dimensional and hierarchical structure, but raises the number of ratings for each dimension from three to five. The five dimensions of the EQ-5D-5L encompass: mobility, self-care, activities of daily living, pain/activities, and pain/tremor. Activities, Pain/Discomfort, and Anxiety/Depression. Individuals select the appropriate level on each dimension based on their condition, creating a five-dimensional description of health status. In addition, the EQ-5D-5L includes a Visual Analog Scale (VAS) that allows individuals to subjectively rate their current state of well-being on a scale of 0 to 100 based on their personal feelings. This score provides a continuous variable to quantify an individual’s overall quality of life.

### 5.6 Ultra brief depression and anxiety screening scale (patient health questionnaire-4, PHQ-4)

Developed by Spitzer et al. (74) for rapid screening of patients for symptoms of depression and anxiety. The scale consists of four questions dealing with symptoms such as lack of interest in daily activities, feelings of restlessness, worry and irritability, and low mood. The scale is rated on a scale of 0 to 2, with a total score ranging from 0 to 8. Based on the total score, symptoms can be categorised as normal, mild, moderate and severe. The Cronbach’s alpha coefficient for the PHQ-4 is 0.833, indicating high internal consistency of the scale (75).

### 5.7 The national institutes of health stroke scale(NIH stroke scale, NIHSS)

The NIHSS scale was developed by the National Institutes of Health in 1989 to objectively and quantitatively assess neurological deficits in stroke patients through standardised examination items (76). Due to its convenience, reliability, effectiveness and comprehensive nature, it has become an indispensable tool in standardised stroke management and clinical research. The scale comprises 11 items with a total score range of 0–42 points, where a higher score indicates more severe neurological impairment. All assessments are conducted by trained neurologists to ensure consistency and accuracy in evaluation.

### 5.8 Indicators of clinical health status

To evaluate the clinical health status of patients, we collected key biochemical and physiological indicators, including blood pressure, HbA1c, FPG, and LDL-C. BP was measured by neurology nurses upon hospital admission and at discharge. The target BP for control was set at <140/90 mmHg, with an ideal range of 120–130/70–80 mmHg; for frail older adults, this target was relaxed to <150/90 mmHg. The target level for LDL-C was <3.36 mmol/L. For glycemic control, HbA1c was used to assess long-term status in diabetic patients, with a target of <7.0%. An HbA1c level of ≥6.5% was used as a diagnostic criterion for diabetes and as a threshold for subgroup analysis. FPG, measured after at least 8 h of fasting, served as an indicator of basal glucose regulation. An FPG level between 6.1–6.9 mmol/L defined impaired fasting glucose (prediabetes) and was also used as a criterion for subsequent subgroup analysis.

### 5.9 Readmission rate

Readmission rate was defined as any unscheduled admission to our hospital within 90 days of the index stroke discharge. To provide a more nuanced understanding of post-discharge outcomes, all readmissions were systematically reviewed and classified into the following predefined categories: Stroke Recurrence: Defined as a new acute neurological deficit, confirmed by neuroimaging (CT or MRI) to be a new ischemic or hemorrhagic lesion, and clinically diagnosed by a neurologist. Other Neurological Complications: Readmissions for direct sequelae of the index stroke, such as post-stroke seizures, symptomatic hydrocephalus, or progressive cerebral edema, that were not classified as a new stroke event. Cardiovascular Events: Readmissions for acute myocardial infarction, unstable angina, heart failure, or arrhythmias. Other Stroke-Related Medical Issues: Readmissions for conditions directly related to stroke management or complications, such as uncontrolled hypertension or hyperglycemia requiring hospitalization, urinary tract infection, or pneumonia. Unrelated Causes: Readmissions for reasons clearly unrelated to the stroke or its management (e.g., orthopedic injury, elective surgery). The primary outcome was the 90-day all-cause readmission rate, calculated as the total number of patients readmitted for any reason divided by the total number of discharged patients. Secondary outcomes included the readmission rates for each specific category listed above, particularly the rate of stroke recurrence.

### 5.10 Statistical analysis

All participants will be analyzed in the group to which they were randomly assigned, regardless of their adherence to the intervention or whether they completed the study. For participants who are lost to follow-up, we will use multiple imputation to handle missing outcome data, under the assumption that data are missing at random. This approach minimises bias and preserves the benefits of randomization. Data will be entered using Excel and statistically analysed using SPSS27.0 software. The data of count data were expressed as frequency and rate; the data of measurement data conforming to normal distribution were described as mean ± standard deviation, and those not conforming to normal distribution were expressed as median (M) and quartile (P25, P75). Count data were analysed using the chi-square test to analyse the differences between the two groups. Prior to inter-group comparisons, univariate analyses were conducted: independent samples t-tests or one-way ANOVA were employed for data conforming to a normal distribution, while the Mann–Whitney U test or Kruskal-Wallis H test was utilized for non-normally distributed data. Paired t-test was used for intra-group comparisons, and repeated-measures ANOVA was used to evaluate the trend of changes in each index, with *p* < 0.05 as the test level.

## 6 Study management

### 6.1 Data collection

Prior to the formal commencement of the study, all investigators will receive systematic training and pass a competency assessment to ensure accuracy and consistency of data collection. The investigators are responsible for recording all clinical observations and laboratory test results in detail on a pre-designed subject registration form, this includes accurate registration and coding of the study subjects to minimise the dropout rate during the follow-up period. In order to maintain data comparability between the intervention and control groups, the time points of the survey and questionnaire content will be designed to be essentially the same. The researcher will ensure that each section of the registration form is completed correctly, and each completed submission will need to be dated and signed in order to validate and retrospectively trace the data at the end of the study. During the data analysis phase, a double-checking mechanism will be implemented to improve the accuracy of data entry. In addition, the research team will consult a statistical expert to select the most appropriate statistical method for analysis.

### 6.2 Storage and archiving of data

Investigators are required to archive all trial data, including lists of subject identification codes, source data and investigator documentation, and records of related correspondence in a study-specific database. All source data and related documents of the study will be archived after the completion of the trial in accordance with legal and regulatory requirements to ensure long-term preservation and traceability of the data.

### 6.3 Ethics and regulations

All procedures of this trial, including conduct, evaluation and documentation, will follow the ethical principles set out in the Good Clinical Practice (GCP) for pharmaceutical clinical trials and the Declaration of Helsinki. The trial will be conducted in strict accordance with local laws and regulations and the protocol has been approved by the Ethics Committee of the Linyi City Central Hospital in Shandong Province (LCH-LW-2025031).

### 6.4 Ethics committee

To ensure the quality of the study, the trial will report the progress of the study to the Ethics Committee on a regular basis and submit revised protocols for approval when necessary. The Ethics Committee has the right to request suspension or termination of the trial if any problem arises during the study.

### 6.5 Informed consent

All participants will voluntarily sign an informed consent form after fully understanding the purpose, methods, potential risks and benefits of the study. For patients who are unable to sign the informed consent form, the option of verbal consent will be offered and it will be ensured that they understand the study and agree to participate.

### 6.6 Confidentiality

Patients’ personal information will be strictly protected and used only for research purposes. The research team will take the necessary steps to ensure data security, including the use of encryption and limiting data access.

### 6.7 Investigator responsibilities

The study leader will oversee the entire study process and ensure that all investigators comply with the trial protocol and study standards. Investigators are required to ensure the authenticity and integrity of the data and maintain high professional and ethical standards during the study.

### 6.8 Approval of trial protocol and revisions

Any revisions to the trial protocol must be reviewed by the study leader and approved by the ethics committee before implementation. The investigator will document all revisions and ensure that all team members are aware of and follow the updated trial protocol.

### 6.9 Data monitoring

The Data Monitoring Committee (DMC) will consist of at least two experts independent of the study team who will regularly monitor the study data, assess the validity and safety of the study and report to the Ethics Committee. The DMC will have the authority to recommend the suspension or termination of the trial if it finds that the study is deviating from the approved protocol or if there are problems.

### 6.10 Safety

All serious adverse events (SAEs) and adverse events (AEs) reported by patients or detected by investigators during the study will be recorded and monitored. All SAEs and AEs must be reported to the Principal Study Leader within 24 h of their occurrence and notified to the Monitor and Ethics Committee in accordance with established procedures. The study leader will be responsible for ensuring that all SAEs and AEs are fully documented and that appropriate measures are taken to protect patient safety and rights. Given that digital interventions may present unique challenges, we systematically monitored unintended consequences at the non-clinical level, including: difficulties or frustrations encountered when using the AI-HEALS platform (such as navigation issues, slow loading times, or unclear operational guidance); increased anxiety, stress, or feelings of being overwhelmed stemming from the educational content or the technical use itself; any evidence suggesting patients misunderstood educational information or used the system in unintended ways, which could lead to confusion or erroneous health behaviors. To capture these effects, research assistants proactively telephoned intervention group participants weekly to enquire about any technical difficulties or negative emotions they might have encountered. Participants were encouraged to report any adverse experiences via the research helpline at any time. All reported unintended consequences were documented, with the research team conducting weekly reviews to identify patterns and implement timely solutions. This enabled the provision of additional technical support to patients or clarification of educational content.

## 7 Discussion

Although previous research has demonstrated the effectiveness of protective motivation theory (PMT) in improving health behaviors in patients with chronic diseases, with relevant studies having been demonstrated in breast cancer prevention (77) and promotion of human papillomavirus women’s health (78), its application to secondary prevention medication adherence in patients with ischaemic stroke has not yet been systematically explored. PMT drives behavioral change through threat assessment (e.g., perceived recurrence risk) and coping assessments (e.g., self-efficacy) to drive behavior change. The medication literacy model, on the other hand, focuses on the patient’s ability to access, understand, and apply medication information, emphasising functional, communicative, critical, and computational skills (79). The present study combines these two theories to construct a ‘motivation-competence-building-behavior consolidation’ intervention framework, and our approach may have stronger theoretical advantages than the limitations of a single theory in complex medication scenarios. This integrative model is similar to that of the ‘behavioral-competence’ dual-pathway intervention strategy (59, 60), which provides a new perspective for breaking the bottleneck of medication adherence in secondary stroke prevention.

Traditional mobile health (mHealth) interventions often face challenges such as fragmented information, lack of systematicity, and insufficient doctor-patient interactions. The AI-HEALS overcomes these barriers by: precise knowledge push, based on a knowledge base vetted by clinicians and pharmacists to ensure scientific and authoritative information about diseases and medicines, and to reduce patients’ medication errors due to misinformation on the Internet; Dynamic interaction support, AI intelligent Q&A system parses patients’ questions in real time and provides personalised medication guidance, similar technology has been proven to be effective in improving medication literacy in asthma patients (52); full-cycle management, relying on the WeChat platform to achieve seamless connection from in-hospital to out-of-hospital, which is in line with China’s ‘Internet+’ healthcare policy guidance, and is particularly suitable for stroke patients who need long-term management. It is suitable for stroke patients who need long-term management. Compared with previous studies, such as smartphone apps (31) or smart pillboxes (80), the AI-HEALS not only enhances immediate interactivity, but also improves multiple dimensions of patients’ medication literacy through structured educational content, which promotes changes in health behaviors such as medication adherence.

Despite the relative rigour of our study design, some potential shortcomings require attention. Our inclusion criteria explicitly required patients to be proficient in the use of WeChat, but older adults or low-education groups may face barriers to operation. For this reason, our nurses will train patients face-to-face on the operating system at the beginning of the intervention to ensure that each subject is proficient. Our study is proposed to be implemented in Linyi City Central Hospital, Shandong Province, and sample representativeness may be limited. The generalizability of our intervention protocol may need to be verified by a multicentre trial in the future. A key limitation of this study is its single-blind design, necessitated by ethical and technical constraints. While this design risks performance bias, as staff were aware of group allocations, we mitigated this through standardized procedures. Furthermore, despite rigorous measures—including physical separation, staff training, and process management—to prevent intergroup “contamination,” we cannot entirely preclude patients encountering information about the AI-HEALS system outside the clinical setting. Such contamination would likely dilute the observed treatment effect, meaning our findings should be considered a conservative estimate. Future studies could address this by employing stricter isolation designs, such as cross-centre randomisation or the Zelen design. Due to the time constraints of the subject, although we proposed to collect data at baseline and at 3 and 6 months of follow-up, this follow-up period is still not long enough for assessing long-term efficacy, especially for a chronic disease such as stroke, and relevant studies have shown that medication adherence in stroke patients declines significantly after 1 year (43). We suggest that follow-up studies could be extended to 12 months or longer and track medication refill rates or hospitalisation rates in conjunction with health insurance data to improve the credibility of the findings. In addition, although the development and maintenance of the AI-HEALS does not require excessive funding, the updating of the Q&A knowledge base and pushed content still requires continuous follow-up by professionals. This is still a challenge for healthcare workers who are busy with their own work, and the stability of the system (e.g., server response speed) may affect the patient experience, but we have found that in the early days of the system’s creation, the stability and the corresponding speed will be gradually optimised as the AI technology advances. How to ensure that this technology can be applied within a wider range of diseases is one of the issues to be addressed in the future. As detailed in recent scoping reviews, the majority of AI applications to date have concentrated on enhancing acute diagnosis—for instance, through automated lesion segmentation and large vessel occlusion detection—and on predicting clinical outcomes (81). These tools are invaluable for rapid decision-making in the hyper-acute window. In contrast, our intervention operates in the post-acute and chronic phases of care, targeting a critical but less technologically addressed domain: secondary prevention. By providing personalized, theory-driven education, our AI system complements diagnostic and predictive models, directly empowering patients to manage their health and mitigate the risk of recurrence. This highlights a complementary and essential role for AI in extending the continuum of intelligent stroke care beyond the hospital setting.

The AI-HEALS in this study is expected to provide significant support for secondary stroke prevention in China, which will reduce the repetitive workload of healthcare professionals through semi-automated education, thus alleviating the pressure on healthcare human resources. According to the China Health Statistics Yearbook, the ratio of neurologists to patients in China may be as low as 1:2,000, especially when high-quality medical resources are mainly concentrated in tertiary hospitals, the AI-HEALS will be expected to be widely promoted in township and village hospitals with its low-cost capital requirements, providing a boost to improve the uneven distribution of medical resources. It is expected to improve medication adherence of relevant patients and reduce stroke recurrence rate, which in turn will reduce family care costs and social health expenditure. Since 2014, China has become one of the countries with the highest direct medical costs for acute ischaemic stroke (82). Therefore, the health economic benefits of this study deserve further in-depth analysis.

The goal of this study was to construct an innovative intervention programme for secondary prevention medication adherence in ischemic stroke patients by integrating the theory of protective motivation with the medication literacy model and with the help of the AI-HEALS. The rationality and validity of the theoretical framework, the precision of the technological platform, and the adaptability of the implementation strategy of this programme will set a new benchmark for behavioral intervention research in chronic diseases. If the results of the trial are positive, it is expected that this disease intervention model will be expanded to other diseases that require long-term medication use, such as coronary heart disease and diabetes mellitus, to promote the application of the ‘motivation-competence’ dual-driven paradigm of health management in a wider range of disease management.
