# Supplementary material for: An intervention study on the secondary prevention medication adherence of ischemic stroke patients based on the protection motivation theory combined with medication literacy education in the AI-HEALS: a randomized controlled trial protocol
Source: Front Public Health. 2026 Jan 29;13:1677253. doi: 10.3389/fpubh.2025.1677253 (PMC12894376; doi:10.3389/fpubh.2025.1677253)
Supplement: Supplementary file 1 [file Supplementary_file_1.doc]

| ****Protocol for Medication Adherence Intervention in Stroke Patients During Hospitalization and Follow-up**** | | | | | | |
| --- | --- | --- | --- | --- | --- | --- |
| ****Time Phase**** | ****Location**** | ****PMT Dimensions**** | ****Medication Literacy Sub-dimensions**** | ****Intervention Objectives**** | ****Intervention Content**** | ****Intervention Modalities**** |
| ****Day 1**** | **Neurology Ward** | **Information Source** |  | **①Establish a personal profile for the patient, collect baseline data, and assess their knowledge and skill level regarding medication.**  ②**Introduce the AI-HEALS system as a tool for accessing medication information and managing health.** | ****Face-to-Face:****  Neurology nurses conduct routine admission education, inquire about the patient's existing knowledge and skills regarding medication, and understand the channels and methods through which the patient obtains drug information.  ****Cognitive Acquisition:**** The HEALS system pushes articles related to stroke medications, helping patients understand different ways and methods of accessing stroke medication information. It also aids patients in learning how to comprehend and evaluate drug information.  **AI-Powered Q&A System**:**** Provide patients with the ability to consult on medication issues anytime and anywhere. The system analyzes and responds based on the content of the patient's inquiries, helping patients better understand drug information. | **Questionnaire Survey；Individualized Instruction；**  **AI-Powered Q&A System；**  **HEALS Platform** |
| ****Day 2**** | **Neurology Ward** | **Perceived Severity, Perceived Susceptibility** | **Comprehension** | **①Enhance patients' awareness of the severity and susceptibility of stroke recurrence.**  **②Increase patients' understanding of the consequences of medication errors and poor adherence.**  **③Help patients comprehend the importance of drug information.** | ****Face-to-Face:**** Neurology nurses conduct education on stroke disease and thrombolysis knowledge, explaining the severity and susceptibility of stroke, as well as the consequences of medication errors. Emphasize the importance of drug information in preventing and treating diseases.  ****Medication Error Case Analysis:**** The HEALS system pushes articles analyzing cases of medication errors, helping patients understand the consequences of such errors.  ****AI Smart Q&A System:**** Provide patients with the ability to consult on medication issues anytime and anywhere. The system analyzes and responds based on the content of the patient's inquiries, helping patients better understand the consequences of medication errors. | **Individualized Instruction；**  **AI-Powered Q&A System；**  **HEALS Platform** |
| ****Day 3**** | **Neurology Ward** | **Internal/External“Rewards”** | **Communication**  **Assessment** | **①Help patients recognize the "benefits" and "sense of pleasure" associated with poor medication adherence.**  **②Strengthen patients' motivation to adopt healthy behaviors.**  **③Assist patients in learning how to communicate medication information with others.**  **④Cultivate patients' ability to critically evaluate drug information.** | ****Face-to-Face:**** "Medical-Pharmaceutical-Nursing" joint rounds, where pharmacists are responsible for explaining medication knowledge, nurses provide behavioral guidance, and physicians review clinical safety.  ****Medication Explanation:**** Distribute self-made oral medication information cards and explain each piece of drug information one by one. Analyze the benefits and barriers to medication adherence.  ****Medication Adherence Analysis:**** The HEALS system pushes articles analyzing the benefits and barriers to medication adherence, helping patients understand the advantages and challenges of adhering to their medication regimen.  ****Information Exchange:**** The HEALS system pushes articles related to peer medication communication, helping patients learn how to exchange medication information with others.  ****Critical Evaluation of Information:**** The HEALS system pushes articles on critical evaluation of drug information. It also educates patients on how to use the AI-powered Q&A system to verify drug information, helping them learn how to critically evaluate drug information. | **Medical-Pharmaceutical-Nursing Joint Guidance；HEALS Platform；AI-Powered Q&A System** |
| ****Day 4**** | **Neurology Ward** | **Self-Efficacy, Response Efficacy** | **Computation** | **①Enhance patients' awareness of correct medication use and a healthy lifestyle.**  **②Improve patients' self-efficacy and confidence in adopting healthy behaviors.**  **③Assist patients in learning how to calculate drug dosage and administration times.**  。 | ****Face-to-Face:**** Attending physicians and nurses emphasize the benefits of correct medication use and a healthy lifestyle, reinforcing intervention confidence. They also explain how to calculate drug dosage and administration times.  ****Medication Calculation:**** The HEALS system pushes articles introducing drug dosage calculations. It also educates patients on how to use the AI-powered Q&A system for drug dosage calculations, thereby enhancing their ability to calculate medication properly. | **Individualized Instruction；HEALS Platform；AI-Powered Q&A System** |
| ****Day 5**** | **Neurology Ward** | **Perceived Costs** | **Comprehension**  **Acquire** | **①Analyze and address the barriers to improving functional drug literacy and poor medication adherence.**  **②Enhance patients' awareness of the consequences of medication errors.**  **③Help patients overcome difficulties in accessing and understanding drug information.** | ****Face-to-Face:**** The attending physician communicates with the patient to jointly analyze the reasons for poor medication adherence and provides feedback. Understand the difficulties patients face in accessing and understanding drug information, and offer assistance.  ****Medication Adherence:**** The HEALS system pushes articles on cases of poor medication adherence. It also educates patients on how to use the AI-powered Q&A system to learn about the correct ways to take their medications, helping them understand the significance of medication adherence in disease prevention. | **Individualized Instruction；HEALS Platform；AI-Powered Q&A System** |
| ****Day 6/Discharge Day**** | **Neurology Ward** | **Coping Potential** | **Communication**  **Assessment** | **①Prevent incorrect behaviors such as wrong medication intake, missed doses, and misinterpretation of drug information, which contribute to poor medication adherence after discharge.**  **②Guide patients on how to safely and effectively use medications after discharge.**  **Help patients develop effective coping strategies for addressing issues encountered during the medication process.**  **Cultivate patients' ability to critically evaluate drug information.** | ****Face-to-Face:**** The attending physician conducts a phase summary, evaluates the patient's mastery of medication skills, reviews the safety of the medication plan, and provides personalized guidance. Researchers assist patients in developing a health behavior plan, helping them set goals for achieving healthy behavior changes. Patients are also guided on how to communicate medication information with others and critically evaluate drug-related information.  ****Post-Discharge Medication Adherence:**** The HEALS system pushes articles on cases of poor medication adherence and again instructs patients on how to use the AI-powered Q&A system to understand the correct way to take medications. This helps patients recognize the importance of medication adherence in disease prevention. | **Individualized Instruction；HEALS Platform；Questionnaire Survey** |
| **Discharge (Second Week of the Study)** | | | | | | |
| **Second Sunday after the start of the study** | **After discharge from the hospital** | **Coping Potential** | **Acquire**  **Comprehension**  **Communication**  **Assessment** | **①Focus on how to correctly and effectively take medications and maintain healthy behaviors without reminders or supervision from healthcare professionals.**  **②Continuously motivate patients to maintain the ability to use medications correctly and effectively.**  **③Help patients consolidate their medication literacy, enabling them to independently acquire, understand, communicate, evaluate, and calculate drug information in daily life.**  **④Provide encouragement to patients who perform well based on log feedback, and offer individualized analysis and guidance to those with poor performance.** | ****Medication Behavior Monitoring and Feedback:**** The HEALS system monitors patients' medication behaviors and provides feedback to help them understand their medication usage.  ****Introduction to Dimensions of Medication Literacy (Acquisition, Understanding, Communication, Evaluation, and Calculation):**** The HEALS system pushes articles related to medication literacy, helping patients consolidate their knowledge in acquiring, understanding, communicating, evaluating, and calculating drug information.  ****Emotion Management Skills Article Push:**** For patients facing difficulties in adherence, articles on emotion management skills are pushed to help them adjust their mindset and overcome challenges. | **Individualized Instruction；HEALS Platform；**Post-Discharge Follow-up**** |
| Repeat the study procedures from the second week onwards until the end of the third month  During the first month - Patients return to the hospital for a follow-up visit, where measurements are conducted | | | | | | |
| Three months after discharge (Based on patient preference, either in-hospital measurements or online questionnaire surveys will be conducted for follow-up.) | | | | | | |
| ****Handling of Special Cases：****  Three consecutive days of missed behavior log uploads and incomplete health plan：  The technical staff in the background will retrieve the patient profile, identify the reasons for their failure to complete the health plan and log uploads, and then conduct telephone follow-ups. Through these follow-ups, they can provide targeted interventions and adjustments to better support the patient. | | | | | | |
